# Associations Between Injury of the Parieto-Insular Vestibular Cortex and Changes in Motor Function According to the Recovery Process: Use of Diffusion Tensor Imaging

**DOI:** 10.3389/fneur.2021.740711

**Published:** 2021-11-03

**Authors:** Seo Yoon Park, Sang Seok Yeo, Sung Ho Jang, In Hee Cho, Seunghue Oh

**Affiliations:** ^1^Department of Physical Therapy, College of Health Sciences, Dankook University, Cheonan, South Korea; ^2^Department of Physical Medicine and Rehabilitation, College of Medicine, Yeungnam University, Daegu, South Korea; ^3^Department of Health, Graduate School, Dankook University, Cheonan, South Korea; ^4^Department of Physical Therapy, Yeungnam University College, Daegu, South Korea

**Keywords:** vestibular compensation, parieto-insular vestibular cortex (PIVC), diffusion tensor imaging-fiber tractography, motor function recovery, continuity

## Abstract

**Background and Purpose:** Parieto-insular vestibular cortex (PIVC) injury can cause symptoms such as abnormal gait and affects the integration and processing of sensory inputs contributing to self-motion perception. Therefore, this study investigated the association of the vestibular pathway in the gait and motor function recovery process in patients with PIVC injury using diffusion tensor imaging (DTI).

**Methods:** We recruited 28 patients with stroke with only PIVC injury and reconstructed the PIVC using a 1.5-T scanner for DTI. Fractional anisotropy (FA), mean diffusivity (MD), and tract volume were measured. The functional ambulatory category (FAC) test was conducted, and motricity index (MI) score was determined. These were conducted and determined at the start (phase 1), end of rehabilitation (phase 2), and during the follow-up 6 months after onset.

**Results:** Although the tract volume of PIVC showed a decrease in subgroup A, all of DTI parameters were not different between two subgroups in affected side (*p* > 0.05). The results of MI and FAC were significantly different according to the recovery process (*p* < 0.05). In addition, FA of the PIVC showed a positive correlation with FAC in phase 2 of the recovery process on the affected side. On the unaffected side, FA of the PIVC showed a significant negative correlation with MI in all processes (*p* < 0.05).

**Conclusion:** The degree of projection pathways to PIVC injury at onset time seems to be related to early restoration of gait function. Moreover, we believe that early detection of the projection pathway for PIVC injury using DTI would be helpful in the clinical evaluation and prediction of the prognosis of patients with PIVC injury.

## Introduction

The parieto-insular vestibular cortex (PIVC) is an ascending pathway that controls equilibrium in the human vestibular system (1, 2). The PIVC is located in the posterior parietal operculum/retroinsular region and extends into posterior sections (2–5). This area is the core region for integrating and processes vestibular and somatosensory information in the cerebral cortex when the head and body positions change. In particular, the PIVC is involved in the processing of self-motion perception, estimation of verticality, processing of visual motion, and motion coherent with gravitational vector (6, 7). Self-motion recognition is essential for monitoring body movements, especially balance and gait. Several studies have reported that PIVC injury can cause symptoms such as extremity weakness/obesity, imbalance, and abnormal gait. In addition, pathology of the PIVC affects the integration and processing of sensory inputs from the vestibular, visual, and somatosensory systems, contributing to self-motion perception (8, 9).

Although previous studies have reported that patients with PIVC injury have an impairment of motor function, no studies have reported an association between tract injury and recovery of motor function in PIVC. However, several studies reported on the relationship between the degree of damage to the corticospinal tract (CST) and the recovery of motor function (10–13). In 2016, Kumar et al. reported that the integrity of CST could predict the recovery process of upper limb motor function (13). In a previous study, diffusion tensor imaging (DTI) was used to assess the degree of CST injury and demonstrated a correlation between the motor impairment at each phase (acute: 3–7 days, subacute: 30 days, and chronic: 90 days) of ischemic stroke. The degree of tract damage and the motor National Institutes of Health Stroke Scale score showed a negative correlation for each phase, and DTI-derived CST assessment could become a marker of motor impairment level (10). Therefore, previous studies suggested that the degree of early tract injury could be used as an indicator to predict motor function recovery.

DTI quantifies diffusion in multiple directions and enables anatomical structures to be visualized using imaging water diffusion patterns (14, 15). Recently, studies have reconstructed PIVC in three dimensions, and several studies have reported on the location and function of the PIVC. However, no study has assessed the relationship between motor function associated with PIVC injury. Given the importance of the PIVC in gait and motor function, demonstrating the relationship of recovery following PIVC injury could provide useful information for the rehabilitation field.

In the current study, we investigated the association of the vestibular pathway with changes in the gait and motor function recovery process in patients with PIVC injury using DTI.

## Materials and Methods

### Subjects

**Figure 1:**
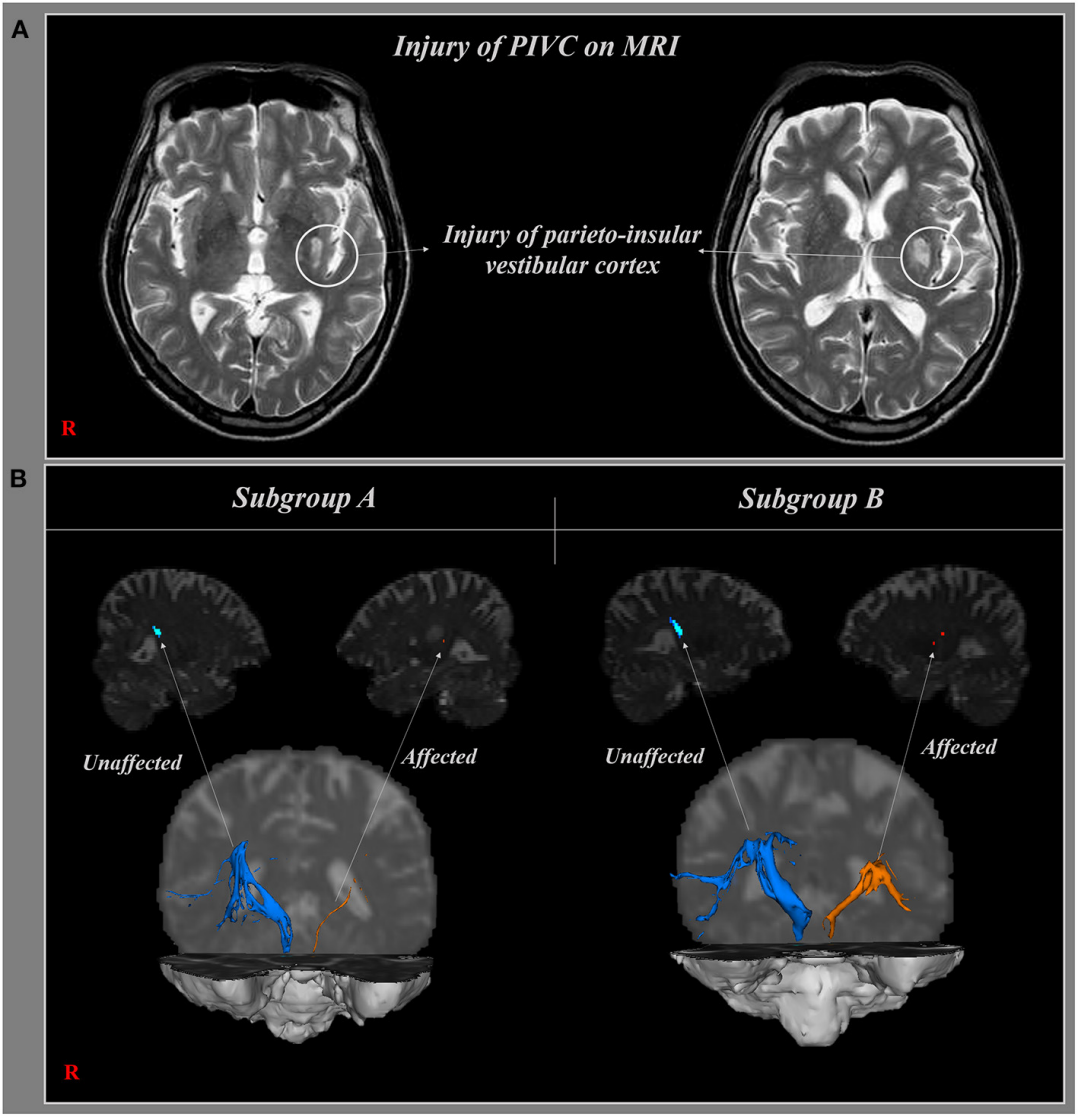
**(A)** Brain magnetic resonance imaging (MRI) shows injury of the projection pathway to parieto-insular vestibular cortex (PIVC). **(B)** Reconstructed projection pathways to PIVC in subgroup **(A,B)**; the pathway was affected due to stroke (orange), and was not affected (blue).

Thirty-six patients with stroke (16 male, 20 female; mean age 63.03 ± 12.06 years) with only PIVC injury on magnetic resonance imaging were recruited for this study at Yeungnam University Hospital (Figure 1A). Inclusion criteria were as follows: (1) first ever stroke, (2) no brain injury due to traumatic injury, (3) PIVC injury due to infarction and hemorrhage, (4) hemiparesis at the time of DTI scanning, and (5) capable of undergoing the functional evaluation. Eight patients were excluded from this study because they were not capable of undergoing functional evaluation. The patients were classified into the following two subgroups based on the continuity of the projection pathway at PIVC injury level on DTI: subgroup A, 16 patients with a discontinuous projection pathway to the PIVC, and subgroup B, 12 patients with a continuous projection pathway to the PIVC at PIVC injury level. A summary of the demographic features according to subgroup A and subgroup B is presented in Table 1. All patients provided informed consent before undergoing DTI and the functional evaluation. The study was approved by the institutional review board of Dankook University (DKU 2020-07-009).

**Table 1:** Demographic features according to subgroup A and subgroup B.

	**No**	**Age (year)**	**Sex (male/female)**	**Month of onset**	**Affected hemisphere (Rt/Lt)**
Subgroup A	1	71	F	27	Rt
	2	74	M	42	Rt
	3	68	F	35	Lt
	4	46	M	46	Rt
	5	79	F	33	Lt
	6	79	F	30	Rt
	7	56	M	33	Lt
	8	54	M	31	Lt
	9	51	F	34	Lt
	10	78	M	31	Lt
	11	67	F	18	Rt
	12	73	F	46	Lt
	13	50	F	42	Lt
	14	33	M	35	Lt
	15	50	M	34	Lt
	16	46	M	37	Rt
	Mean values	60.94 (14.34)	8/8	34.63 (7.13)	6/10
Subgroup B	1	76	F	33	Lt
	2	72	M	32	Rt
	3	55	M	50	Lt
	4	68	M	37	Rt
	5	71	F	38	Rt
	6	68	M	73	Rt
	7	74	F	49	Lt
	8	33	M	23	Lt
	9	56	F	53	Rt
	10	70	F	93	Lt
	11	66	F	64	Rt
	12	60	M	37	Lt
	Mean values	64.08 (11.87)	6/6	48.50 (19.96)	6/6

*(± standard deviation); M, male; F, female*.

### Diffusion Tensor Image

Acquisition of DTI data was performed using a six-channel head coil on a 1.5 T Philips Gyro scan Intera (Philips, Best, The Netherlands) with single-shot echo-planar imaging. For each of the 32 non-collinear diffusion sensitizing gradients, 67 contiguous slices were collected parallel to the anterior commissure-posterior commissure line. The imaging parameters were as follows: acquisition matrix = 96 × 96; reconstructed matrix = 192 × 192; field of view = 240 × 240 mm^2^; TR = 10,726 ms; TE = 76 ms; parallel imaging reduction factor (SENSE factor) = 2; EPI factor = 49; b = 1,000 s/mm^2^; NEX = 1; and a slice thickness of 2.5 mm with no gap (acquired voxel size 1.3 × 1.3 × 2.5 mm^3^) (16, 17).

### Probabilistic Fiber Tracking

The diffusion-weighted imaging data was analyzed using the Oxford Centre for Functional Magnetic Resonance Imaging of the Brain (FMRIB) Software Library (FSL; www.fmrib.ox.ac.uk/fsl). Affine multi-scale two-dimensional registration was used to correct the head motion effect and image distortion due to eddy current. Fiber tracking used a probabilistic image method based on a multifiber model, and performed in this study utilizing image routines implemented in FMRIB Diffusion (5,000 streamline samples, 0.5 mm step lengths, curvature thresholds = 0.2) (18).

**Figure 2:**
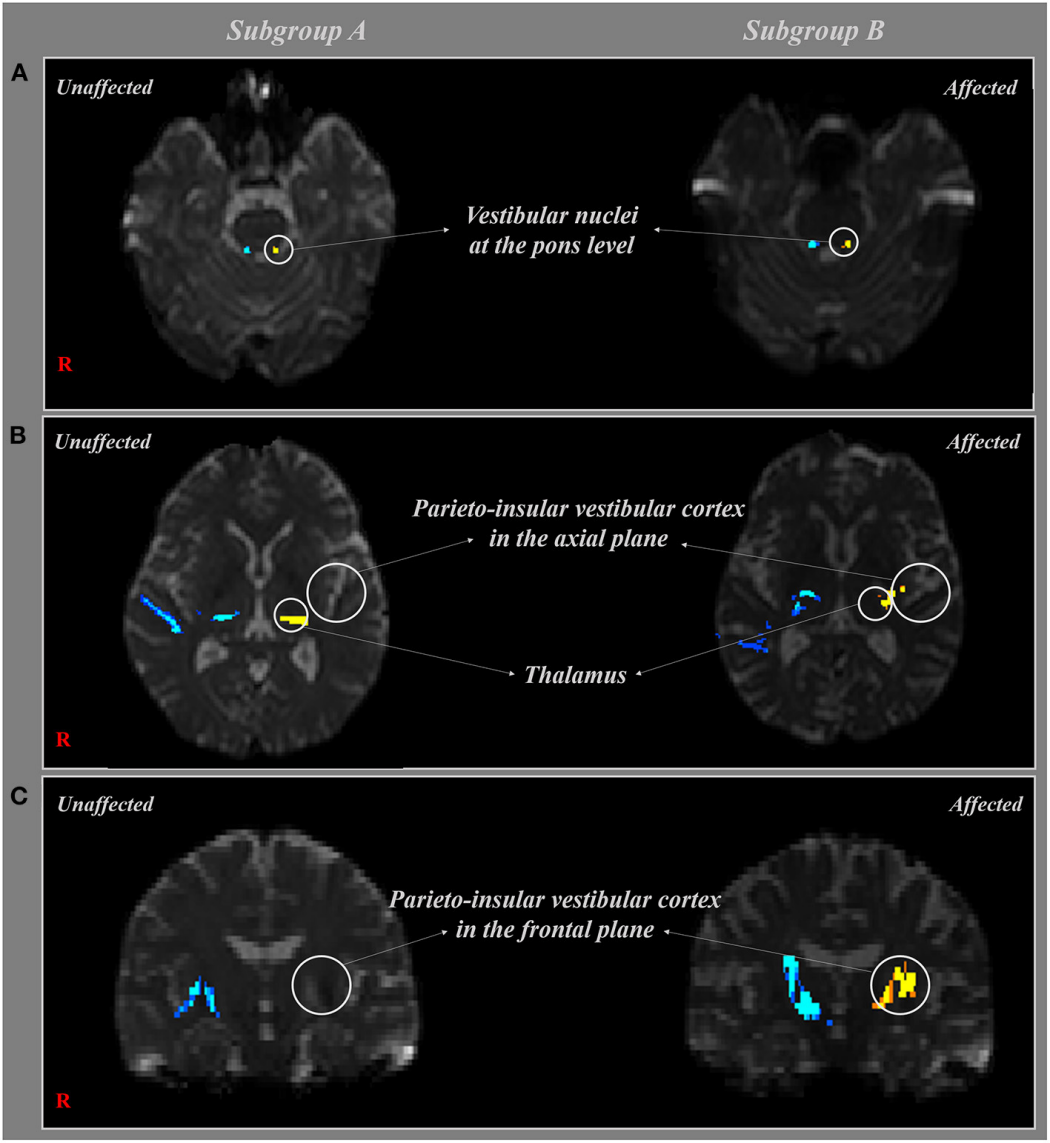
The projection pathway to parieto-insular vestibular cortex (PIVC) is shown at each level. The affected side (yellow and red) and unaffected side (blue) are shown in two subgroups according to the type of diffusion tensor imaging (DTI) tractography. **(A)** The projection pathway to PIVC that passes by the vestibular nuclei at the pons level, **(B)** thalamus and PIVC in the axial plane. **(C)** The projection pathway to PIVC that passes by the PIVC in the frontal plane.

The projection pathway to the core vestibular cortex was determined by selection of fibers passing through seed and two target regions of interest (ROI). The seed ROI and target ROI of the projection pathway to PIVC were determined as follows: seed ROI—the vestibular nuclei at the level of pons equivalent to Deiters' nuclei and Schwalbe's nuclei, and target ROI—two of the target ROI were placed on posterior parietal operculum and thalamus (Figure 2) (19). The 5,000 samples were generated from the seed voxel, and the results were visualized at the threshold of 1 streamline through each voxel for analysis. The values of fractional anisotropies (FA), mean diffusivities (MD), and tract volume (voxel number) of the projection pathway to PIVC were measured.

### Functional Evaluation

This study measured the ability of motor function using the functional ambulatory category (FAC) test and motricity index (MI) score with well-established reliabilities and validities (20, 21). Functional evaluation was conducted at the start of rehabilitation (phase 1, 13.21 ± 5.12 days), end of rehabilitation (phase 2, 40.57 ± 15.47 days), and at follow-up 6 months after onset (follow-up). The MI score was used to assess the motor function of the affected upper and lower extremities (maximum score: 100) (22). The FAC test was used to evaluate ambulatory function (23). FAC was designed to examine the levels of assistance and included six ordinal and hierarchical categories: 0 (non-ambulatory), 1 (needs continuous support from one person to help in carrying weight), 2 (needs intermittent support from one person to help with balance), 3 (needs only verbal supervision), 4 (help is required on stairs and uneven surfaces), and 5 (can walk independently anywhere).

### Statistical Analysis

SPSS software (ver. 20.0; SPSS, Inc., Chicago, IL, USA) was used to analyze the results. Independent *t*-tests were used to compare the significant differences in DTI parameters after injury between subgroups A and B. Two-way repeated measures analysis of variance (ANOVA) was used to compare the significant differences in functional evaluation according to the recovery process between the two groups. The non-parametric Spearman correlation analysis was used to investigate the association between PIVC injury in DTI scanning and motor function according to the recovery process. The level of statistical significance was set at *p* < 0.05 (uncorrected).

## Results

### Diffusion Tensor Image

The results of DTI parameters between subgroups A and B were as follows. On the affected side, there were no significant differences in the mean FA and MD values of the PIVC between subgroups A and B (*p* > 0.05). Although the tract volume of the reconstructed projection pathway to the PIVC was less in subgroup A than in subgroup B, there were no significant differences between the two subgroups (*p* > 0.05) (Figures 1B, 2). In addition, no DTI parameters were significantly different between the two subgroups on the unaffected side (*p* > 0.05) (Table 2).

**Table 2:** Comparison of DTI parameters in PIVC between subgroup A and subgroup B.

		**Subgroup A**	**Subgroup B**	***p***
Affected	FA	0.38 (0.16)	0.42 (0.05)	0.346
	MD	0.70 (0.28)	0.75 (0.07)	0.511
	Tract volume	215.69 (172.39)	443.17 (434.73)	0.067
Unaffected	FA	0.46 (0.04)	0.45 (0.03)	0.600
	MD	0.87 (0.07)	0.82 (0.09)	0.092
	Tract volume	411.69 (300.53)	458.58 (359.51)	0.718

*Values represent mean (± standard deviation)*.

*DTI, diffusion tensor imaging; PIVC, parieto-insular vestibular cortex; Affected, affected hemisphere; Unaffected, unaffected hemisphere; FA, fractional anisotropy; MD, mean diffusivity*.

^*^ p < 0.05.

### Functional Evaluations

The results of MI and FAC between subgroups A and B according to the recovery process are shown in Table 3. The results of the two-way repeated measures ANOVA indicated that there was no significant difference between subgroups A and B according to the recovery process (interaction time × group) in MI and FAC, respectively (*F* = 0.359, *p* = 0.620; *F* = 0.504, *p* = 0.571). In the case of the main effect of time, the results of MI and FAC were significantly different according to the recovery process (*F* = 37.032, *p* < 0.001; *F* = 70.596, *p* < 0.001, respectively).

**Table 3:** Comparison of the results of MI and FAC between subgroup A and subgroup B according to recovery process.

		**Subgroup A**	**Subgroup B**	**Time**		**Interaction (Time** **×** **Group)**	
				***F***	***p***	***F***	***p***
MI	Phase 1	40.92 (23.33)	50.81 (23.48)	37.032	<0.001^*^	0.359	0.620
	Phase 2	61.29 (13.79)	66.50 (12.86)				
	Follow-up	66.11 (13.32)	72.58 (15.51)				
FAC	Phase 1	1.06 (1.12)	1.67 (1.23)	70.596	<0.001^*^	0.504	0.571
	Phase 2	2.63 (1.09)	3.50 (1.00)				
	Follow-up	3.44 (3.92)	3.92 (0.90)				

*Values represent mean (± standard deviation)*.

*MI, motricity index; FAC, functional ambulatory category; Phase 1, start of rehabilitation; Phase 2, end of rehabilitation; Follow-up, follow-up 6 months after onset*.

* *p < 0.05*.

### Correlation According to the Recovery Process

**Figure 3:**
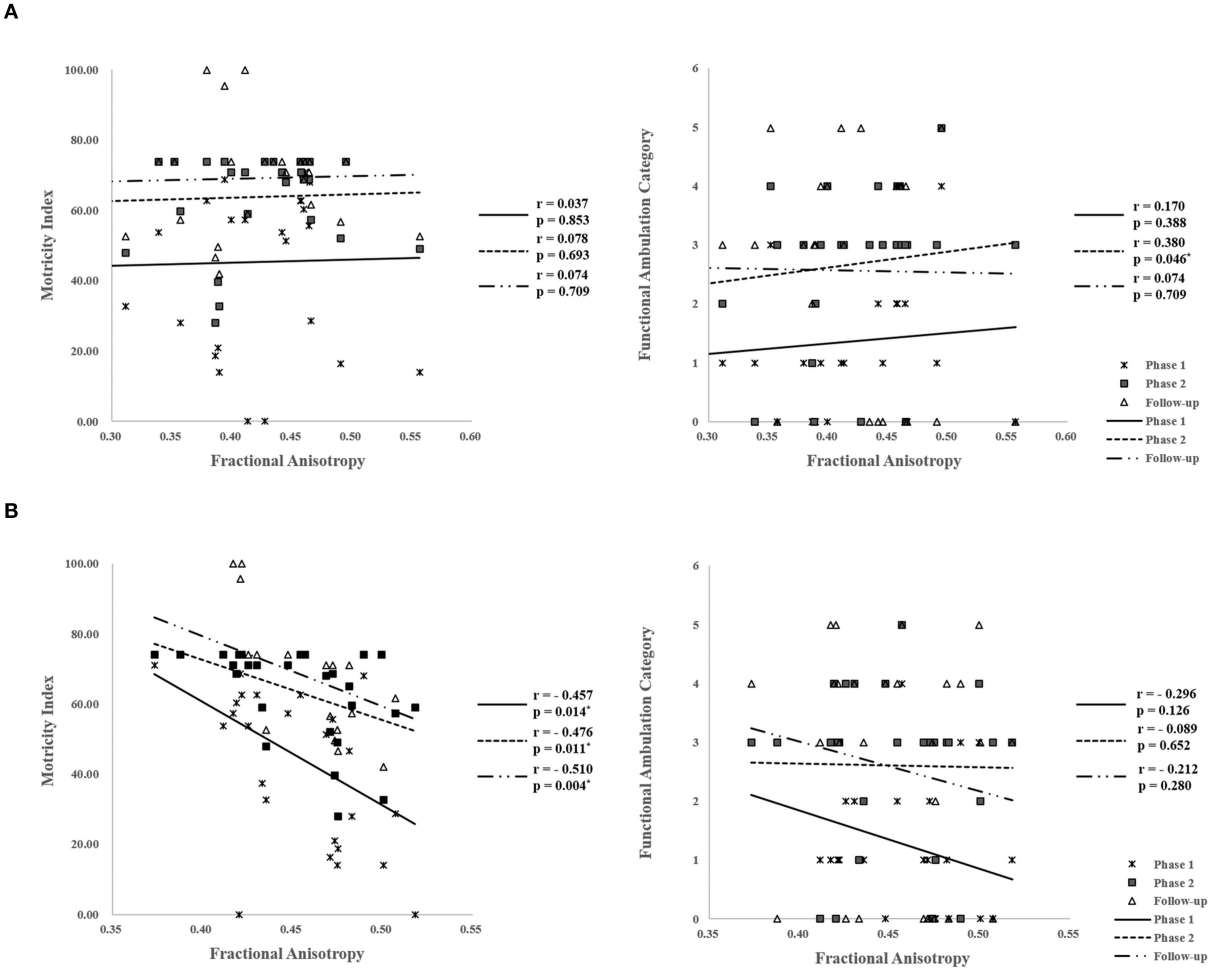
**(A)** Affected hemisphere. Correlations between motor function and fractional anisotropy (FA) of diffusion tensor imaging (DTI) parameters in the affected hemisphere according to recovery process. **(B)** Unaffected hemisphere. Correlations between motor function and fractional anisotropy (FA) of diffusion tensor imaging (DTI) parameters in the unaffected hemisphere according to recovery process. *p < 0.05.

A summary of the correlation between DTI parameters of the reconstructed projection pathway to the PIVC and functional evaluations according to the recovery process is shown in Tables 4, 5. On the affected side, the mean FA of the PIVC showed a positive correlation with FAC in phase 2 of the recovery process (*r* = 0.466, *p* = 0.012). Conversely, the mean FA values of PIVC were not correlated with phase 1 (*r* = 0.282, *p* = 0.146) and during follow-up (*r* = 0.113, *p* = 0.566) (Figure 3A). In addition, there was no correlation between the mean FA of DTI and the results of MI (*p* > 0.05). All DTI parameters except for FA values were also not significantly correlated with functional evaluations (*p* > 0.05) (Table 4). On the unaffected side, the mean FA values of the PIVC were significantly negatively correlated with MI in phase 1 (*r* = −0.457, *p* = 0.014), phase 2 (*r* = −0.476, *p* = 0.011), and during follow-up (*r* = −0.510, *p* = 0.004) (Figure 3B). The mean MD values of the PIVC were significantly negatively correlated with FAC in phase 2 (*r* = −0.434, *p* = 0.021). On the other hand, there was no correlation between mean FA values of DTI and FAC (*p* > 0.05). As with the affected side, all DTI parameters except for FA and MD values in the unaffected side were not significantly correlated with functional evaluations (*p* > 0.05) (Table 5).

**Table 4:** Correlation between motor function and DTI parameters of PIVC in the affected hemisphere according to recovery process.

		**Affected**			**MI**			**FAC**		
		**FA**	**MD**	**Tract volume**	**Phase 1**	**Phase 2**	**Follow-up**	**Phase 1**	**Phase 2**	**Follow-up**
Affected	FA	1								
	MD	**0.845** **(0.000)**	1							
	Tract volume	0.104 (0.599)	0.278 (0.152)	1						
MI	Phase 1	0.037 (0.853)	0.048 (0.808)	0.099 (0.618)	1					
	Phase 2	0.078 (0.693)	0.077 (0.696)	0.151 (0.444)	**0.711** **(0.000)**	1				
	Follow-up	0.056 (0.776)	0.102 (0.605)	0.209 (0.286)	**0.676** **(0.000)**	**0.822** **(0.000)**	1			
FAC	Phase 1	0.282 (0.146)	0.025 (0.901)	−0.164 (0.404)	**0.788** **(0.000)**	**0.612** **(0.001)**	**0.490** **(0.008)**	1		
	Phase 2	**0.466** **(0.012)**	0.043 (0.828)	0.079 (0.689)	**0.413** **(0.029)**	**0.579** **(0.001)**	**0.544** **(0.003)**	**0.448** **(0.017)**	1	
	Follow-up	0.113 (0.566)	−0.084 (0.671)	0.060 (0.762)	**0.454** **(0.015)**	**0.600** **(0.001)**	**0.614** **(0.001)**	**0.398** **(0.036)**	**0.760** **(0.000)**	1

*DTI, diffusion tensor imaging; PIVC, parieto-insular vestibular cortex; Affected, affected hemisphere; MI, motricity index; FAC, functional ambulatory category; Phase 1, start of rehabilitation; Phase 2, end of rehabilitation; Follow-up, follow-up 6 months after onset; FA, fractional anisotropy; MD, mean diffusivity*.

*Values represent Spearman correlation coefficient (p value)*.

*Correlations significant at p < 0.05 are in bold (uncorrected)*.

**Table 5:** Correlation between motor function and DTI parameters of PIVC in the unaffected hemisphere according to recovery process.

		**Unaffected**			**MI**			**FAC**		
		**FA**	**MD**	**Tract volume**	**Phase 1**	**Phase 2**	**Follow-up**	**Phase 1**	**Phase 2**	**Follow-up**
Unaffected	FA	1								
	MD	−0.216 (0.270)	1							
	Tract volume	−0.010 (0.958)	0.118 (0.551)	1						
MI	Phase 1	**−0.457** **(0.014)**	−0.088 (0.658)	0.141 (0.473)	1					
	Phase 2	**−0.476** **(0.011)**	−0.143 (0.467)	0.254 (0.192)	**0.711** **(0.000)**	1				
	Follow-up	**−0.510** **(0.006)**	0.080 (0.687)	0.232 (0.236)	**0.676** **(0.000)**	**0.822** **(0.000)**	1			
FAC	Phase 1	−0.300 (0.121)	−0.263 (0.176)	0.002 (0.991)	**0.788** **(0.000)**	**0.612** **(0.001)**	**0.490** **(0.008)**	1		
	Phase 2	−0.085 (0.667)	**−0.434** **(0.021)**	0.063 (0.751)	**0.413** **(0.029)**	**0.579** **(0.001)**	**0.544** **(0.003)**	**0.448** **(0.017)**	1	
	Follow-up	−0.220 (0.261)	−0.093 (0.639)	0.000 (1.000)	**0.454** **(0.015)**	**0.600** **(0.001)**	**0.614** **(0.001)**	**0.398** **(0.036)**	**0.760** **(0.000)**	1

*DTI, diffusion tensor imaging; PIVC, parieto-insular vestibular cortex; Unaffected, unaffected hemisphere; MI, motricity index; FAC, functional ambulatory category; Phase 1, start of rehabilitation; Phase 2, end of rehabilitation; Follow-up, follow-up 6 months after onset; FA, fractional anisotropy; MD, mean diffusivity*.

*Values represent Spearman correlation coefficient (p value)*.

*Correlations significant at p < 0.05 are in bold (uncorrected)*.

## Discussion

In the current study, we investigated the recovery process of motor function according to the degree of PIVC injury through a follow-up study for 6 months from onset. For this, we reconstructed the projection pathway to the PIVC as the core vestibular cortex in onset time and measured the motor function using FAC and MI from phase 1 to follow-up. We found that in phase 2, the FAC score was higher in subgroup B (patients with the continuous projection pathway to the PIVC) than in subgroup A (patients with the discontinuous projection pathway to the PIVC). In the correlation analysis, on the affected side, only FA values of projection pathways to the PIVC in onset time showed a mild positive correlation with FAC in phase 2. The FA value indicates the degree of directionality of water diffusion (24, 25). It represents white matter organization, and increased FA is related to increased myelination (25–27). We assumed that the increased FA values without change in tract volumes and MD values seem to indicate increased integration of neural fibers. Hence, the degree of projection pathways to PIVC injury in onset time seems to be related to the degree of recovery regarding gait function until the subacute stage. However, on the unaffected side, the FA values of the PIVC showed a significant negative correlation with MI from phase 1 to follow-up. Neuroplasticity is induced through hyperactivation of the PIVC tract without restoration of motor function to compensate for the affected side (28–30).

The PIVC is a core region of vestibular input into the cortical regions of the central vestibular system (31–33). In particular, the PIVC is involved in the integration and processing of sensory inputs from the vestibular, visual, and somatosensory systems, contributing to self-motion perception (8, 9, 31–33). Self-motion perception is essential for monitoring body movements, especially balance and walking (33). When vestibular inputs are absent or in conflict owing to an injury of the vestibular cortex region, the brain may generate an inaccurate self-motion perception in these patients (33). Hence, PIVC lesions can cause symptoms such as extremity weakness/numbness, imbalance, and gait abnormalities (17).

In DTI studies, Yeo et al. (34) demonstrated patients with injury of the vestibulospinal tract following lateral medullary syndrome (e.g., loss of consciousness, ataxia, postural instability, confusion, headache, incoordination, and visual deficits) (4). In 2019, Yeo et al. showed the relationship between the vestibular neural pathway, including the projection pathway to the PIVC and the vestibulospinal tract and balance according to aging. Moreover, DTI parameters in the vestibular neural pathway are associated with age-related reductions in balance ability (34). Together, these results commonly show that the PIVC is associated with central vestibular disorders and impairment of body movement, especially balance and gait function because PIVC encodes the vestibular signals contributing to self-motion perception (33). These results are consistent with the current findings showing that the degree of projection pathways for PIVC injury is associated with gait function, which requires complex integration of multisensory input and musculoskeletal systems (35, 36). Consequently, as far as we are aware, this is the first DTI study to demonstrate the degree of PIVC injury related to recovery of motor function from onset to follow-up.

Several previous studies have reported that patients with bilateral or unilateral loss of vestibular function are immediately ataxic, with severe postural instability (30, 37). These studies commonly report that over weeks and months, vestibular function such as postural stability and balance control improves through the process of vestibular compensation using vision, light touch, sensory bio-feedback, and potentiation of remaining vestibular function (30, 37, 38). Moreover, the recovery of postural stability and balance control after the loss of vestibular function is dependent on the remaining vestibular function (39, 40). Thus, we assume that the subgroup A appears to have improved gait function due to vestibular compensation, and subgroup B appears to have improved gait function due to recovery of the remaining vestibular function. Moreover, according to our results, the degree of the remaining projection pathway to the PIVC seems to accelerate the time-course of gait function recovery by using the remaining vestibular function. Conversely, even if the patients with a discontinuous projection pathway to the PIVC showed slow recovery of gait function, it seems that the gait function was eventually restored by vestibular compensation.

In the current study, the FA values of the PIVC were negatively correlated with MI from phase 1 to follow-up. Several studies have reported that persistent motor cortex activity in the unaffected hemisphere has been associated with poor motor function (41–45). In 2010, Kwak et al. reported that changes in the FA value of the unaffected side were not correlated with the motor function of the affected side. The results of the current study appear to be consistent with those of previous studies. Thus, it can be regarded as induced neuroplasticity by the hyperactivation of the PIVC tract without restoration of motor function to compensate for the affected side (28–30).

There are several studies for prediction of motor function using DTI in stroke. In 1999, Yang et al. demonstrated that the diffusion anisotropy ratio (affected/unaffected hemisphere) measured within 12 h of stroke onset correlated with the Barthel index at 3 months after stroke onset in 26 patients with cerebral infarction (46). Subsequently, Gillard et al. (47) reported that information obtained from DTI morphology (white matter distortion) in the early stage (11 h to 2 weeks after onset) of stroke (10 patients) provided useful information for predicting motor outcome at 4 months after onset (47). We believe that the findings of the current study are consistent with the findings of previous studies that attempted to predict motor outcome using a DTI study. Consequently, we believe that early detection of the projection pathway for PIVC injury using DTI would be helpful in clinical evaluation and prediction of prognosis for patients with PIVC injury.

The present study has a number of limitations that warrant consideration. First, we reconstructed the projection pathway to the PIVC only in onset time except phase 1, phase 2, and follow-up. Second, we could not consider other neural tracts related to gait function and balance, such as the vestibulospinal tract, corticoreticular pathway, and corticospinal tract. Third, we could not consider other clinical evaluations to measure the walking and balance function specifically, such as spatiotemporal parameters and the burg balance scale. Another limitation is that DTI analysis is operator dependent, and due to fiber complexity, this may result in false-positive or false-negative results for the fiber track. Further studies would be necessary, including follow-up DTI results and more neural tracts that are related to walking ability.

We investigated the recovery process of motor function according to the degree of PIVC injury through a follow-up study for 6 months from onset. According to the results, the degree of projection pathways to PIVC injury in onset time seems to be related to early restoration regarding gait function. Especially, it was found that the increased FA value of the PIVC in the affected hemisphere in onset time seems to be related to restoration regarding gait function until the subacute stage. Moreover, we believe that early detection of the projection pathway for PIVC injury using DTI would be helpful in the clinical evaluation and prediction of prognosis for patients with PIVC injury.

## Data Availability Statement

The raw data supporting the conclusions of this article will be made available by the authors, without undue reservation.

## Ethics Statement

The studies involving human participants were reviewed and approved by the institutional review board of Dankook University (DKU 2020-07-009). The patients/participants provided their written informed consent to participate in this study.

## Author Contributions

SP and SO: writing—original draft preparation, methodology, and investigation. SY: writing—reviewing and editing, and conceptualization. SJ: data curation and project administration. IC: writing—original draft preparation and visualization. All authors contributed to the article and approved the submitted version.

## Funding

This research was supported by Basic Science Research Program through the National Research Foundation of Korea (NRF), funded by the Ministry of Education, Science and Technology (2021R1A2C1095047).

## Conflict of Interest

The authors declare that the research was conducted in the absence of any commercial or financial relationships that could be construed as a potential conflict of interest.

## Publisher's Note

All claims expressed in this article are solely those of the authors and do not necessarily represent those of their affiliated organizations, or those of the publisher, the editors and the reviewers. Any product that may be evaluated in this article, or claim that may be made by its manufacturer, is not guaranteed or endorsed by the publisher.
